# Determinants of fertility in Rwanda in the context of a fertility transition: a secondary analysis of the 2010 Demographic and Health Survey

**DOI:** 10.1186/1742-4755-11-87

**Published:** 2014-12-13

**Authors:** Vedaste Ndahindwa, Collins Kamanzi, Muhammed Semakula, François Abalikumwe, Bethany Hedt-Gauthier, Dana R Thomson

**Affiliations:** School of Public Health, University of Rwanda, Kigali, Rwanda; Rwanda Biomedical Center, Kigali, Rwanda; National Institute of Statistics of Rwanda, Kigali, Rwanda; Partners in Health, Rwinkwavu, Rwanda; Department of Global Health and Social Medicine, Harvard Medical School, Boston, MA USA

**Keywords:** Fertility, TFR, Rwanda, Africa, DHS

## Abstract

**Background:**

Major improvements to Rwanda’s health system, infrastructure, and social programs over the last decade have led to a rapid fertility transition unique from other African countries. The total fertility rate fell from 6.1 in 2005 to 4.6 in 2010, with a 3-fold increase in contraceptive usage. Despite this rapid national decline, many women still have large numbers of children. This study investigates predictors of fertility during this fertility transition to inform policies that improve individuals’ reproductive health and guide national development.

**Methods:**

We used Poisson regression to separately model number of children born to ever married/cohabitated women (n = 8,309) and never married women (n = 1,220) age 15 to 49 based on 2010 Rwanda Demographic and Health Survey data. We used backward stepwise regression with a time offset to identify individual and household factors associated with woman’s fertility level, accounting for sampling weights, clustering, and stratification.

**Results:**

In ever married/cohabitating women, high fertility was significantly associated (p < 0.05) with the following variables: unmet need for contraception (IRR = 1.07), women’s desire for children (5+ versus 0–2 children: IRR = 1.22), woman’s number of siblings (8–20 versus 0–4: IRR = 1.03), and couples who desired different numbers of children (husband wants more: IRR = 1.04; husband wants fewer: IRR = 1.04). Low fertility in ever married/cohabitating women was associated with women’s education (higher versus no education: IRR = 0.66), household wealth (highest versus lowest quintile: IRR = 0.93), and delayed sexual debut (25+ versus 8–18 years: IRR = 0.49). In never married women, low fertility was associated with education (higher versus no education: IRR = 0.22), household wealth (highest versus lowest quintile: IRR = 0.58), delayed sexual debut (25–49 versus 8–18 years: IRR = 0.43), and having an unmet need for contraception (IRR = 0.69).

**Conclusions:**

Although the study design does not allow causal conclusions, these results suggest several strategies to further reduce Rwanda’s national fertility rate and support families to achieve their desired fertility. Strategies include policies and programs that promote delayed sexual debut via educational and economic opportunities for women, improved access to reproductive health information and services at schools and via health campaigns, and involvement of men in family planning decision making.

## Introduction

Population growth and high fertility rates in resource poor settings can be a challenge for both the society and individuals. A growing population can affect the well-being of that population in terms of socioeconomic development, environmental sustainability, and resource supply [1]. Resource poor countries with population growth are challenged to create jobs for a budding workforce while their governments lack resources to meet increasing demand for services and infrastructure [2]. The effect of high fertility is also challenging for individuals. When many children are born to one mother, there is an economic burden on her household and an increased chance of her family entering into poverty [3]. In families that do not have enough resources for education, food, and health care, children - especially girls - may be forced to drop out of school and to marry early [2]. High fertility also increases the risk that a child is born prematurely or with low birth weight [4] and becomes stunted as she grows [5], and premature birth increases maternal health risks [6].

The “demographic transition” describes a widely observed phenomena whereby a population transitions from high levels of mortality and fertility to low levels of mortality and fertility [7]. This transition is typified by an initial drop in child morality due to improved infrastructure, health system developments, and socioeconomic improvements followed by a decrease in fertility rates years later. While countries in sub-Saharan Africa have been slow to enter and pass through the fertility transition, Rwanda has been an exception [8]. Rapid improvements to the health system, infrastructure, and social programs over the last decade have launched Rwanda into a rapid fertility transition. Between 2005 and 2010, the mortality rate among children under five was halved from 152 to 76 deaths per 1000 live births, marking one of the fastest improvements in child mortality in human history [9]. The average fertility rate in Rwanda dropped from 6.1 births per woman to 4.6 births per woman in 2010 after the percent of women using a modern method of contraception increased from 17% in 2005 to 52% in 2010 [10]. In other East African countries the fertility decline is still low: in Uganda fertility declined between 2006 and 2011, from 6.7 children per woman to 6.2 children [11]; in Kenya fertility dropped from 4.9 in 200 to 4.6 in 2008 [12] while in Tanzania the fertility was 5.7 in 2004–05 and slightly declined to 5.4 births per woman in 2010 [13].

This drop in fertility and uptake of contraception in Rwanda coincides with a major shift in attitudes by government officials about family planning as it related to economic development policies. Faced with the reality that Rwanda has the highest population density of any country in Africa (416 persons per square kilometer with an annual population growth rate of 2.6% [14]), smaller families and limited population growth became priorities for individual well-being as well as national progress. Officials subsequently launched widespread campaigns to shift public attitudes toward acceptance of small families with an informal goal of bringing the total fertility rate to less than 4 children per woman [15]. Following the implementation of mandatory free primary education, and in response to the rising cost of living, the government has performed sensitization campaigns to encourage couples to have only as many children as the family can afford to feed, educate, and care for. This has been reinforced at the community-level by community health workers and community leaders in monthly “community works” meetings. Despite this major shift in fertility, there are many families in Rwanda still having large families; more than 20% of women between 15 and 49 currently have had five or more births [16].

Rwanda is in an important demographic transition that is setting a course for the country’s economic development and bucking trends in a region of slow fertility transition. Understanding predictors of fertility can support the development of policies and interventions that both support families to achieve their desired fertility and inform government economic policies and infrastructure development plans. Results may also inform fertility policies and programming elsewhere in sub-Saharan Africa. This article examines some of the determinants of fertility rates in Rwanda, looking separately at women who have ever been married/cohabitating and women who have never been married.

## Methods

This analysis is based on the data collected from the 2010 Rwanda Demographic and Health Survey (RDHS). The RDHS is a nationally representative two-stage cluster sample designed to provide population and health indicators at national, province and district levels. Villages were the primary sampling unit (PSU) with 413 rural PSUs and 79 urban PSUs sampled, stratified by district. Twenty-six households were sampled per PSU [16]. Women provided informed consent before participating in the survey; we were granted permission by the MEASUREDHS Project to use these de-identified data for this analysis.

A total of 13,671 women age 15 to 49 years participated in the 2010 RDHS. Women who never had sex were excluded from the analysis. Among women who reported ever having sex, the analyses were stratified by the 8,309 women currently or ever married/cohabitating and the 1,220 women who had never married.

The main outcome, level of fertility, is defined as the total number of children ever born to women in the childbearing period (15–49 years). The predictor variables were mainly the proximate determinants of fertility - current marital status, age at first cohabitation, age at first sexual intercourse, age at first birth, use of contraceptives - and socio-demographic variables that may predict fertility in Rwanda. Twenty variables in the 2010 RDHS were identified as potential predictors of fertility.

We developed a multivariable Poisson regression model with a natural logarithmic link function to assess associations between predictor variables and fertility rates. The offset term in the Poisson model was set to natural log of current age of the woman. The modeling development involved two stages. Potential predictors were identified in bivariate analyses. Variables that were differentially distributed across ever married or never married women with different numbers of children were retained based on a chi-squared test (p < 0.05). We tested remaining variables for collinearity at the r > 0.5 level, and excluded the variable that was more weakly associated with fertility. Multivariable models were built using backward stepwise regression considering ever married and never married women separately. Variables that were significantly associated with fertility at the 95% confidence level were retained, and all models were adjusted for age, province, and urban/rural residence. Adjusted incidence rate ratios (IRR) with 95% confidence intervals (CI) are reported to assess the association of fertility with the demographic characteristics (age, number of siblings, number of unions), socioeconomic characteristics (education, wealth, religion, residence, types of earnings), geography (province), fertility behavior and desires (age at first sex, unmet need for family planning, ideal number of children) and, among ever married women, husband’s desire for children. All analyses were performed in Stata v12 with svyset statements to apply sampling weights, and account for clustering and stratification.

## Results

The bivariate results for determinants of fertility and the number of children ever born is presented for women who were ever married/cohabitated in Table 1, and for women who have never married in Table 2. Five percent of ever married/cohabitated woman had no children, 45.7% had 1 to 3 children, and 49.3% had more than 3 children. All potential predictors considered in the bivariate analyses were significant for this group. For never married women, 49.6% had no children, 48.3% had 1 to 3 children, and 2.1% had more than three children. All considered covariates were significant in the bivariate analyses except urban/rural residence and religion (p = 0.101 and p = 0.386, respectively).

**Table 1:** **Percentage of ever married**/**cohabiting women by number of children and various characteristics**

	Total children ever born						
	No child		1-3 children		4+ children		p-value
	%	95% CI	%	95% CI	%	95% CI	
**Overall (n = 8,309)**	**5.0**	**[4.5-5.4]**	**45.7**	**[44.6-46.9]**	**49.3**	**[48.2-50.5]**	
**Age in 5-year groups**							
15-19 (n = 100)	38.9	[29.6-49.1]	61.1	[50.9-70.4]	0.0	--	<0.001
20-24 (n = 1,079)	15.9	[13.8-18.2]	82.9	[80.5-85.0]	1.3	[0.8-2.1]	
25-29 (n = 1,931)	4.9	[4.1-6.0]	79.8	[77.9-81.6]	15.3	[13.6-17.1]	
30-34 (n = 1,662)	2.5	[1.8-3.3]	41.8	[39.2-44.5]	55.7	[52.9-58.5]	
35-39 (n = 1,364)	1.9	[1.3-2.8]	22.3	[20.0-24.9]	75.7	[73.2-78.1]	
40-44 (n = 1,092)	1.9	[1.2-2.9]	15.8	[13.8-18.1]	82.3	[79.9-84.5]	
45-49 (n = 1,081)	1.3	[0.8-2.2]	11.6	[9.8-13.7]	87.0	[84.8-89.0]	
**Province**							
Kigali city (n = 1,017)	7.3	[5.9-9.2]	59.4	[55.8-62.9]	33.3	[29.5-37.2]	<0.001
South (n = 2,061)	4.8	[4.0-5.8]	45.0	[42.8-47.2]	50.2	[48.0-52.5]	
West (n = 1,897)	4.5	[3.7-5.5]	45.2	[42.7-47.7]	50.3	[47.8-52.8]	
North (n = 1,327)	5.3	[4.2-6.6]	42.5	[40.0-45.1]	52.2	[49.6-54.8]	
East (n = 2,007)	4.3	[3.5-5.4]	43.4	[41.1-45.7]	52.3	[50.0-54.6]	
**Type of place of residence**							
Urban (n = 1,297)	7.7	[6.4-9.2]	54.9	[51.5-58.3]	37.5	[34.0-41.0]	<0.001
Rural (n = 7,012)	4.5	[4.1-5.0]	44.3	[43.1-45.5]	51.2	[50.0-52.4]	
**Highest educational level**							
No education (n = 1,773)	3.1	[2.4-4.0]	31.4	[29.2-33.6]	65.5	[63.3-67.7]	<0.001
Primary (n = 5,628)	5.3	[4.7-5.9]	49.1	[47.7-50.5]	45.6	[44.3-47.0]	
Secondary (n = 768)	6.4	[4.8-8.4]	50.8	[46.6-55.0]	42.8	[38.5-47.2]	
Higher (n = 140)	9.6	[5.6-16.1]	72.8	[62.7-80.9]	17.6	[10.8-27.4]	
**Type of earnings from respondent's work**							
Not paid, in-kind only (n = 2,068)	5.7	[4.7-6.8]	42.7	[40.6-44.9]	51.6	[49.5-53.7]	<0.001
Cash only, or cash and in-kind (n = 5,425)	4.4	[3.8-5.0]	46.2	[44.8-47.7]	49.4	[47.9-50.8]	
Not currently working (n = 816)	7.1	[5.5-9.1]	50.2	[46.2-54.3]	42.7	[38.8-46.7]	
**Wealth index**							
Poorest (n = 1,766)	3.8	[2.9-4.9]	45.2	[42.8-47.7]	51.0	[48.7-53.4]	<0.001
Poorer (n = 1,687)	4.4	[3.5-5.5]	44.7	[42.4-47.1]	50.8	[48.5-53.1]	
Middle (n = 1,613)	5.0	[4.1-6.3]	44.8	[42.3-47.3]	50.2	[47.6-52.7]	
Richer (n = 1,576)	5.3	[4.3-6.5]	42.0	[39.4-44.5]	52.7	[50.2-55.2]	
Richest (n = 1,667)	6.4	[5.3-7.7]	52.4	[49.9-54.9]	41.2	[38.5-43.9]	
**Religion**							
Catholic (n = 3,483)	4.5	[3.8-5.3]	42.9	[41.1-44.7]	52.7	[50.8-54.5]	<0.001
Protestant (n = 3,355)	5.6	[4.9-6.5]	48.2	[46.3-50.1]	46.2	[44.4-48.0]	
Adventist (n = 1,176)	4.7	[3.6-6.2]	47.7	[44.6-50.9]	47.5	[44.5-50.6]	
Muslim (n = 129)	5.7	[2.7-11.7]	49.9	[41.8-58.0]	44.4	[36.1-53.0]	
Other (n = 166)	2.3	[0.9-5.8]	38.7	[31.4-46.6]	59.0	[51.0-66.5]	
**Unmet need for spacing/limiting**							
Not currently in union (n = 617)	3.5	[2.3-5.3]	46.5	[42.6-50.5]	50.0	[46.0-54.0]	<0.001
Unmet need (n = 1,488)	1.0	[0.6-1.7]	38.6	[36.0-41.2]	60.4	[57.8-62.9]	
No unmet need (n = 5,115)	6.0	[5.3-6.7]	50.5	[49.1-51.9]	43.6	[42.1-45.0]	
Infecund, menopausal (n = 1,084)	6.2	[4.9-7.8]	33.1	[30.1-36.2]	60.7	[57.4-63.9]	
**Number of siblings of respondent**							
0-4 (n = 1,830)	6.4	[5.4-7.6]	49.9	[47.7-52.2]	43.6	[41.4-45.9]	<0.001
5-7 (n = 3,841)	5.1	[4.4-5.9]	45.8	[44.1-47.4]	49.1	[47.5-50.8]	
8-20 (n = 2,633)	3.7	[3.1-4.5]	42.7	[40.7-44.7]	53.6	[51.5-55.6]	
**Ideal number of children**							
0-2 children (n = 1,666)	9.6	[8.2-11.2]	54.5	[52.0-57.0]	35.9	[33.4-38.4]	<0.001
3 Children (n = 2,680)	6.8	[5.9-7.7]	59.5	[57.6-61.5]	33.7	[31.9-35.6]	
4 children (n = 2,446)	2.4	[1.8-3.1]	40.1	[38.0-42.2]	57.5	[55.4-59.6]	
5+ children (n = 1,415)	0.5	[0.2-1.0]	20.5	[18.5-22.8]	79.0	[76.7-81.0]	
**Husband's desire for children**							
Married, both want same (n = 3,958)	5.9	[5.2-6.8]	50.5	[48.9-52.1]	43.6	[41.9-45.2]	<0.001
Married, husband wants more (n = 700)	3.3	[2.2-5.0]	40.5	[36.7-44.4]	56.2	[52.3-60.0]	
Married, husband wants fewer (n = 1,182)	2.4	[1.7-3.5]	46.0	[43.1-48.9]	51.6	[48.7-54.5]	
Married, don't know (n = 899)	8.5	[6.9-10.4]	34.8	[31.7-38.1]	56.7	[53.4-59.9]	
No longer living together (n = 1,475)	3.0	[2.2-3.9]	43.3	[40.8-45.8]	53.7	[51.2-56.2]	
**Number of unions**							
One (n = 7,245)	5.4	[4.9-5.9]	48.0	[46.8-49.3]	46.6	[45.3-47.8]	<0.001
More than one (n = 1,056)	2.1	[1.4-3.2]	29.9	[27.2-32.8]	68.0	[65.1-70.7]	
**Age at first sex**							
8-18 (n = 3,827)	2.9	[2.4-3.4]	38.7	[37.0-40.5]	58.4	[56.7-60.1]	<0.001
19-24 (n = 3,523)	5.7	[4.9-6.5]	50.5	[48.8-52.2]	43.8	[42.1-45.5]	
25-49 (n = 788)	13.3	[11.1-15.7]	59.6	[55.9-63.3]	27.1	[23.8-30.7]

**Table 2:** **Percentage of never married women by number of children and various characteristics**

	Total children ever born						
	No child		1-3 children		4+ children		p-value
	%	95% CI	%	95% CI	%	95% CI	
**Overall (n = 1,220)**	**49.6**	**[46.5-52.7]**	**48.3**	**[45.3-51.3]**	**2.1**	**[1.4-3.1]**	
**Age in 5-year groups**							
15-19 (n = 332)	77.0	[71.8-81.5]	23.0	[18.5-28.2]	0.0	--	<0.001
20-24 (n = 462)	50.4	[45.2-55.6]	49.6	[44.4-54.8]	0.0	--	
25-29 (n = 243)	28.8	[23.2-35.2]	69.8	[63.5-75.4]	1.4	[0.4-4.3]	
30-34 (n = 78)	39.5	[29.0-51.1]	54.5	[43.1-65.5]	5.9	[2.2-15.0]	
35-39 (n = 48)	12.1	[5.1-26.1]	76.1	[61.5-86.4]	11.9	[5.3-24.4]	
40-44 (n = 41)	18.9	[10.0-32.6]	60.2	[45.1-73.5]	21.0	[11.0-36.4]	
45-49 (n = 16)	13.5	[3.3-41.5]	67.4	[40.9-86.1]	19.0	[6.2-45.8]	
**Province**							
Kigali city (n = 284)	57.9	[51.6-64.0]	40.7	[34.6-47.1]	1.4	[0.4-4.4]	0.001
South (n = 281)	37.8	[31.9-44.2]	58.1	[51.8-64.1]	4.1	[2.3-7.1]	
West (n = 229)	55.5	[48.3-62.4]	43.2	[36.5-50.2]	1.3	[0.4-3.8]	
North (n = 170)	50.3	[42.0-58.6]	47.4	[39.8-55.1]	2.4	[0.9-5.8]	
East (n = 256)	48.8	[42.7-55.0]	49.9	[43.9-56.0]	1.2	[0.4-3.8]	
**Type of place of residence**							
Urban (n = 328)	54.2	[48.5-59.7]	44.8	[39.3-50.5]	1.0	[0.3-3.1]	0.101
Rural (n = 892)	48.2	[44.5-51.9]	49.3	[45.7-52.9]	2.4	[1.6-3.6]	
**Highest educational level**							
No education (n = 124)	27.6	[20.3-36.2]	67.1	[58.2-74.9]	5.3	[2.5-11.0]	<0.001
Primary (n = 810)	48.2	[44.5-51.8]	49.8	[46.2-53.4]	2.0	[1.2-3.3]	
Secondary (n = 238)	60.6	[54.0-66.9]	38.6	[32.3-45.2]	0.8	[0.2-3.1]	
Higher (n = 48)	85.0	[71.8-92.7]	15.0	[7.3-28.2]	0.0	--	
**Type of earnings from respondent's work**							
Not paid, in-kind only (n = 282)	52.8	[46.4-59.1]	44.8	[38.4-51.3]	2.4	[1.2-5.1]	<0.001
Cash only, or cash and in-kind (n = 718)	43.4	[39.5-47.3]	54.0	[50.2-57.8]	2.6	[1.6-4.1]	
Not currently working (n = 220)	66.3	[58.6-73.2]	33.7	[26.8-41.4]	0.0	--	
**Wealth index**							
Poorest (n = 199)	36.0	[29.6-42.9]	58.9	[52.1-65.4]	5.1	[2.8-9.2]	<0.001
Poorer (n = 181)	36.9	[29.9-44.5]	60.3	[52.4-67.7]	2.8	[1.2-6.6]	
Middle (n = 213)	50.1	[42.8-57.3]	48.5	[41.3-55.8]	1.4	[0.5-4.3]	
Richer (n = 196)	53.9	[46.4-61.2]	44.0	[37.0-51.3]	2.1	[0.8-5.5]	
Richest (n = 431)	60.5	[54.9-65.8]	39.0	[33.7-44.6]	0.5	[0.1-2.1]	
**Religion**							
Catholic (n = 529)	46.5	[42.0-51.1]	51.1	[46.6-55.6]	2.4	[1.4-4.1]	0.384
Protestant (n = 510)	52.7	[48.1-57.3]	44.8	[40.3-49.3]	2.5	[1.4-4.4]	
Adventist (n = 138)	51.2	[42.9-59.4]	48.8	[40.6-57.1]	0.0	--	
Muslim (n = 22)	46.0	[27.8-65.4]	54.0	[34.6-72.2]	0.0	--	
Other (n = 21)	44.8	[24.9-66.5]	55.2	[33.5-75.1]	0.0	--	
**Ideal number of children**							
0-2 children (n = 564)	49.8	[45.0-54.6]	49.5	[44.7-54.3]	0.7	[0.2-1.8]	0.003
3 Children (n = 467)	48.6	[43.9-53.3]	49.2	[44.7-53.8]	2.2	[1.2-4.0]	
4 children (n = 158)	53.1	[44.6-61.4]	40.8	[33.0-49.2]	6.1	[3.2-11.2]	
5+ children (n = 27)	48.5	[29.3-68.1]	45.0	[26.0-65.5]	6.5	[1.6-23.0]	
**Unmet need for spacing/limiting**							
Not sexually active (n = 949)	53.4	[49.9-56.9]	45.1	[41.7-48.6]	1.5	[0.9-2.5]	<0.001
Unmet need (n = 70)	59.4	[47.7-70.2]	37.1	[26.7-48.9]	3.4	[0.9-12.6]	
No unmet need (n = 168)	20.3	[14.3-27.9]	74.3	[66.6-80.7]	5.4	[2.8-10.3]	
Infecund, menopausal (n = 33)	68.1	[51.5-81.1]	31.9	[18.9-48.5]	0.0		
**Number of siblings of respondent**							
0-4 (n = 367)	51.2	[45.6-56.7]	46.2	[40.8-51.8]	2.6	[1.3-4.9]	0.014
5-7 (n = 531)	52.6	[48.1-57.2]	46.5	[42.0-51.0]	0.9	[0.4-2.1]	
8-20 (n = 321)	43.2	[37.9-48.6]	53.3	[47.7-58.9]	3.5	[1.9-6.3]	
**Age at first sex**							
8-18 (n = 803)	53.8	[50.1-57.4]	44.0	[40.5-47.6]	2.2	[1.4-3.5]	0.005
19-24 (n = 311)	41.7	[35.5-48.1]	56.4	[50.1-62.5]	2.0	[0.9-4.4]	
25-49 (n = 71)	45.3	[33.8-57.2]	54.7	[42.8-66.2]	0.0	--	

The results of the multivariable analysis are presented in Table 3. These results showed lower fertility among women with more education and with greater household wealth. In ever married/cohabitated women, more education was progressively associated with fewer children; the IRR was 0.96 (95% CI: 0.94-0.98), 0.90 (95% CI: 0.87-0.94) and 0.66 (95% CI: 0.61-0.73) respectively for women with primary, secondary and higher education levels compared to women with no education. In never married women, only women with more than a secondary education had fewer children than women with no education (IRR = 0.22; 95% CI: 0.09-0.52). Household wealth had a stronger effect on limiting fertility among never married women (IRR = 0.58, 95% CI: 0.46-0.63) than ever married/cohabitated women (IRR = 0.93, 95% CI: 0.90-0.96). Working status was associated with higher fertility among never married women; women employed for cash (IRR = 1.34; 95% CI: 1.06-1.70) or in-kind compensation (IRR = 1.38; 95% CI: 1.11-1.71) had more children than unemployed women.

**Table 3:** **Predictors of fertility level in ever married and never married women**, **Rwanda 2010**

	Ever married women*		Never married women*	
Variables	IRR	[95% Conf. Interval]	IRR	[95% Conf. Interval]
**Age in 5**-**year groups**				
15-19	1.00		1.00	
20-24	1.67	[1.39 - 2.01]	1.92	[1.51 - 2.44]
25-29	2.58	[2.14 - 3.11]	2.92	[2.28 - 3.75]
30-34	3.57	[2.96 - 4.30]	2.99	[2.19 - 4.09]
35-39	4.16	[3.45 - 5.01]	4.29	[3.27 - 5.62]
40-44	4.57	[3.79 - 5.51]	3.69	[2.65 - 5.12]
45-49	4.85	[4.02 - 5.87]	3.42	[2.29 - 5.10]
**Highest educational level**				
No education	1.00		1.00	
Primary	0.96	[0.94-0.98]	0.89	[0.75-1.05]
Secondary	0.90	[0.87-0.94]	0.81	[0.63-1.04]
Higher	0.66	[0.61-0.73]	0.22	[0.09-0.52]
**Wealth index**				
Poorest	1.00		1.00	
Poorer	0.97	[0.95-0.99]	0.92	[0.78-1.08]
Middle	0.98	[0.96-1.01]	0.65	[0.54-0.79]
Richer	0.96	[0.94-0.99]	0.69	[0.54-0.88]
Richest	0.93	[0.90-0.96]	0.58	[0.46-0.73]
**Age at first sex**				
8-18	1.00		1.00	
19-24	0.78	[0.76-0.79]	0.79	[0.70-0.90]
25-49	0.49	[0.47-0.52]	0.43	[0.34-0.56]
**Unmet need for spacing/limiting**				
No unmet need	1.00		1.00	
Unmet need	1.07	[1.05-1.09]	0.69	[0.51-0.94]
Infecund, menopausal	0.74	[0.71-0.77]	0.57	[0.39-0.82]
No longer in union, not sexually active	0.99	[0.94-1.05]	0.56	[0.50-0.64]
**Type of earnings**				
Not currently working			1.00	
Cash only, or cash and in-kind			1.34	[1.06-1.70]
Not paid, in-kind only			1.38	[1.11-1.71]
**Ideal number of children**				
0-2 children	1.00			
3 Children	1.05	[1.02-1.08]		
4 children	1.14	[1.10-1.17]		
5+ children	1.22	[1.18-1.25]		
**Husband’s desire for children**				
Married, both want same	1.00			
Married, husband wants more	1.04	[1.01-1.07]		
Married, husband wants fewer	1.04	[1.02-1.07]		
Married, husband doesn’t know	0.98	[0.96-1.01]		
No longer in union	0.87	[0.83-0.91]		
**Number of unions**				
One	1.00			
More than one	0.87	[0.85-0.90]		
**Number of siblings**				
0-4	1.01	[0.99-1.04]		
5-7	1.00			
8-20	1.03	[1.01-1.05]		
N. of observations	8309		1220	

*Adjusted for province and urban/rural residence.

Age of sexual debut was strongly associated with fertility rate; women who were 25 years or older at first sex had less than half the fertility rate as women whose first sex was before age 19 in both ever married/cohabitated women (IRR = 0.49, 95% CI: 0.47, 0.52) and never married women (IRR = 0.43, 95% CI: 0.34, 0.56). In ever married/cohabitated women, having an unmet need for family planning was associated with higher levels of fertility (IRR = 1.07, 95% CI: 1.05-1.09), while unmet need was associated with lower fertility in never married women (IRR = 0.69, 95% CI: 0.51, 0.94).

Several additional factors were associated with fertility in ever married/cohabitated women. In these women, their ideal number of children was associated with fertility level; fertility was 1.22 times higher (95% CI: 1.18,1.25) among women who wanted 5 or more children compared to women who wanted 0 to 2 children. Women whose partners wanted a different number of children had higher fertility than women who wanted the same number of children as their partners (husband wanted more children IRR = 1.04, 95% CI: 1.01-1.07, husband wanted fewer children IRR = 1.04, 95% CI: 1.02-1.07). Ever married/cohabitated women who have been in more than one union had 0.87 times the fertility rate of women who had been in only one union (95% CI: 0.85, 0.90). Women from large families with 8 or more siblings had slightly higher fertility than women from average size families with 5 to 7 siblings (IRR = 1.03, 95% CI: 1.01, 1.05), and women with 4 or fewer siblings had similar fertility as women from average size families.

## Discussion

Although we cannot draw causal conclusions from these results, our study suggests several risk factors for high fertility including having an unmet need for family planning, early sexual debut, limited access to education and economic opportunity for women, valuing larger families over small families, and married couples disagreeing about desired number of children. We suggest several strategies for continuing to reduce fertility, particularly delaying sexual debut though increased educational and economic opportunity for women, increased access to reproductive health knowledge and services in schools and through public campaigns, and involving men in family planning programs and campaigns.

### Delayed sexual debut

The legal age at marriage in Rwanda is 21, in part to encourage delayed sexual debut. Because marriage after 21 is later than many countries, we do not make the typical assumption that women wait until marriage to start having sex, and instead measure age of sexual debut as a predictor of lifetime fertility. Studies which use marriage as a proximate determinant of fertility assume that it reflects sexual activity; our finding that delayed sexual debut is strongly correlated with lower fertility is consistent with this literature [17, 18].

Multiple theories describe our finding that low fertility is associated with advancements in women’s education, higher wealth status, and delayed sexual debut. Through school, educated women receive more messages about delayed sexual debut and delayed marriage, and the values of spaced and limited births, than girls who drop out of school [19]. Educated young women also have increased social power to control their reproductive decisions, access to different types of partners than less educated women, increased exposure to mass media, and more opportunities for professional growth [19]. Educated mothers tend to have greater health literacy and access to financial resources to diagnose and care for sick children, though community health worker programs and mutuelle insurance might mitigate this challenge in Rwanda. A number of government programs aimed at both women’s empowerment and delaying age of sexual debut encourage women’s secondary and higher education through scholarships, campaigns, incentives (such as free laptops), and lowered entry requirements [20–22].

### Improve knowledge and access to reproductive health services

As expected, ever married/cohabitated women with an unmet need for contraception are more likely to have more children. This may be because women who have had several children want to space or limit births [23], though it could also be that women with a current unmet need for family planning are the kinds of women who had mistimed or unplanned pregnancies in the past leading to a larger number of births than women whose contraceptive needs have been met over time.

Conversely, never married women with an unmet need for family planning had fewer children than women whose needs were met. This may reflect that never married women who have already had a child are better linked into reproductive health services and are more likely to use contraceptives regularly and correctly than sexually active women without children. In Rwanda there is a cultural and religious emphasis on abstinence before marriage. Although secondary schools provide sex education, contraceptives are not made readily available to young women, and reproductive health services targeting youth are often linked to HIV testing and counseling [24], which may increase stigma, though this has not been adequately evaluated [25].

Among ever married/cohabitated women, those who desired more children had more children. The causality of this relationship is not clear due to rationalization bias, that is, a woman reports an inflated ideal number of children because she is reluctant to state a number that is smaller than her current number of children [8]. Women from very large families tended to have slightly more children than women from average or small families which may reflect family pressure or norms [26, 27].

### Involvement of men in family planning

Couples who disagreed on their desired number of children had slightly more children than couples who agreed; this pattern was observed whether the wife or the husband wanted more children. A simple explanation for this phenomenon is that in these couples, one partner is placing pressure on the other to have another child. The limited research about men’s involvement in sexual and reproductive health programming worldwide reveals major deficits in reproductive health programs to educate and involve men in family planning dialogues [28]. Most studies focus on the strong desire among men to be involved with family planning decision making [29], and a few evaluate the effectiveness of involving men. A randomized study in Ethiopia, for example, found that husband involvement during home-based family planning education was associated with twice the use of modern contraceptive usage in married women after one year compared to home-based family planning education with the wife only [30]. Couples that report different numbers of desired children might be struggling to communicate effectively about reproductive health which could lead to mistimed and unwanted pregnancies.

### Recommendations

The government’s campaigns to sensitize couples to desire smaller families, and programs that incentivize delayed sexual debut and education appear to have reduced the fertility rate between 2005 and 2010. In addition to current approaches, the government and health care providers should consider coordination between the health and education sectors to make reproductive health education and services available to young people during and after secondary school to prevent mistimed and unplanned pregnancies. Reframing joint family planning and HIV programs as reproductive health services, rather than HIV testing and counselling, may reduce stigma and invite wider utilization of both reproductive health services. Recent initiatives to add youth clinics to local health facilities to be able to answer questions and provide services to young people in a non-judgmental, confidential environment are promising [31].

A Ministry of Health project that provides couples with education and free access to male vasectomy has resulted in over 2000 male vasectomies since 2008, and helps couples to dialogue about reproductive health desires later in their reproductive careers [32, 33]. Further empowering women through education and supporting the involvement of men in reproductive health education and decision making earlier in life may help couples to dialogue effectively about reproductive health and achieve their ideal family size.

### Limitations

There were several limitations to this analysis. First, these results are based on secondary analysis of cross-sectional data so the results represent associations only; we cannot draw conclusions about causes of low and high fertility. Our recommendations make some assumptions about causality based on existing evidence, though should be interpreted with caution. Second, we were not able to explore associations with all potential determinates of fertility because they were not captured in the survey. Induced abortion, for example, is an important determinant of fertility [34] but the DHS questionnaire does not distinguish induced and spontaneous abortions.

## Conclusion

Rwanda is unique in Africa for its sharp declines in desired and actual fertility in recent years. The unique circumstances that led to this decline may provide lessons for other countries in the region. However, despite this fertility decline, the fertility rate remains above the national target of 3 children per woman. The government’s strong and coordinated position to sensitize the public about the benefits of smaller families, promote women’s empowerment through education, and encourage delayed sexual debut through late marriage may have all played roles in the steep decline in fertility. Based on these results we recommend additional programs to improve access to reproductive health services, particularly to young, unmarried women, and involve men in family planning decision making.

## Abbreviations

CI: Confidence Interval
IRR: Incidence Rate Ratio
RDHS: Rwanda Demographic and Health Survey
PSU: Primary Sampling Unit
TFR: Total Fertility Rate.
